# Treatment of Alcohol Withdrawal

**Published:** 1998

**Authors:** Hugh Myrick, Raymond F. Anton

**Affiliations:** Hugh Myrick, M.D., is an assistant professor of psychiatry and Raymond F. Anton, M.D., is a professor of psychiatry at the Medical University of South Carolina, Department of Psychiatry, Center for Drug and Alcohol Programs, Charleston, South Carolina

**Keywords:** AOD withdrawal syndrome, treatment method, inpatient care, outpatient care, symptom, disease severity, alcohol withdrawal agents, drug therapy, delirium tremens, AODR (alcohol and other drug related) seizure, patient assessment, cormorbidity, treatment cost, benzodiazepines, adrenergic receptors, special populations, literature review

## Abstract

Appropriate treatment of alcohol withdrawal (AW) can relieve the patient’s discomfort, prevent the development of more serious symptoms, and forestall cumulative effects that might worsen future withdrawals. Hospital admission provides the safest setting for the treatment of AW, although many patients with mild to moderate symptoms can be treated successfully on an outpatient basis. Severe AW requires pharmacological intervention. Although a wide variety of medications have been used for this purpose, clinicians disagree on the optimum medications and prescribing schedules. The treatment of specific withdrawal complications such as delirium tremens and seizures presents special problems and requires further research.

Symptoms of alcohol withdrawal (AW) may range in severity from mild tremors to massive convulsions (e.g., withdrawal seizures). Mild AW can cause pain and suffering; severe AW can be life-threatening. The goals of AW treatment are to relieve the patient’s discomfort, prevent the occurrence of more serious symptoms, and forestall cumulative effects that might worsen future withdrawals. Withdrawal treatment also provides an opportunity to engage patients in long-term alcoholism treatment.

This article explores the management of AW and co-occurring conditions, evaluates different treatment settings and medications, and addresses considerations in treating special populations.

## Clinical Features of Alcohol Withdrawal

The symptoms of AW reflect overactivity of the autonomic nervous system, a division of the nervous system that helps manage the body’s response to stress. The signs and symptoms of AW typically appear between 6 and 48 hours after heavy alcohol consumption decreases. Initial symptoms may include headache, tremor, sweating, agitation, anxiety and irritability, nausea and vomiting, heightened sensitivity to light and sound, disorientation, difficulty concentrating, and, in more serious cases, transient hallucinations. These initial symptoms of AW intensify and then diminish over 24 to 48 hours.

Delirium tremens (DT’s), the most intense and serious syndrome associated with AW, is characterized by severe agitation; tremor; disorientation; persistent hallucinations; and large increases in heart rate, breathing rate, pulse, and blood pressure. DT’s occur in approximately 5 percent of patients undergoing withdrawal and usually appear 2 to 4 days after the patient’s last use of alcohol.

Seizures occur in up to 25 percent of withdrawal episodes, usually beginning within the first 24 hours after cessation of alcohol use. For more detail on the signs and symptoms of AW, see the article by Trevisan et al., pp. 61–66.

## Supportive Care for Alcohol Withdrawal

Certain medical disorders that commonly co-occur with alcoholism can exacerbate symptoms of AW or complicate its treatment. The purpose of supportive care is to treat such disorders and to remedy nutritional deficiencies. Patients with AW should be subject to a physical examination, with particular emphasis on detecting conditions such as irregular heartbeat (i.e., arrhythmia), inadequate heart function (i.e., congestive heart failure), liver disease (e.g., alcoholic hepatitis), pancreatic disease (i.e., alcoholic pancreatitis), infectious diseases (e.g., tuberculosis), bleeding within the digestive system, and nervous system impairment. Vital signs (e.g., heartbeat and blood pressure) should be stabilized and disturbances of water and nutritional balances corrected.

The presence of water in the blood and within cells is essential for the performance of physiological processes and to maintain both heart and kidney function. Some patients undergoing AW may require intravenous fluids to correct severe dehydration resulting from vomiting, diarrhea, sweating, and fever. Conversely, many AW patients may retain excess water in their blood and tissues. In these patients, intravenous administration of liquid may overload the heart’s ability to pump blood, leading to heart failure. In most cases, water balance can be maintained by oral administration of fluids.

Alcoholics are often deficient in electrolytes, or “minerals” (e.g., magnesium, phosphate, and sodium). Because these substances play a major role in metabolism, electrolyte disturbances may lead to severe and even life-threatening metabolic abnormalities. A causal relationship has been postulated between low magnesium levels and the occurrence of seizures or delirium. Although such an association has not been verified, magnesium supplments may help improve general withdrawal symptoms.

Some alcoholics exhibit vitamin deficiencies, presumably because of poor dietary habits as well as from alcohol-induced changes in the digestive tract that impair the absorption of nutrients into the bloodstream. Two dietary factors of particular importance in AW are folic acid and thiamine. Folic acid plays a role in the synthesis of the cell’s genetic material and maturation of certain blood cells. Folic acid deficiency can lead to changes in blood cells, including a form of anemia. Patients undergoing AW should be administered an oral multivitamin formula containing folic acid for a few weeks.

Thiamine plays an essential role in the body’s energy metabolism. Thiamine deficiency in alcoholics is a factor in the development of Wernicke syndrome, a condition characterized by severe confusion, abnormal gait, and paralysis of certain eye muscles. In addition, Wernicke syndrome can progress to an irreversible dementia. All patients being treated for AW should be given 100 milligrams (mg) of thiamine as soon as treatment begins and daily during the withdrawal period.^1^ Supplies of thiamine stored in the body are limited even in the absence of alcoholism. Therefore, thiamine should always be administered before giving an alcoholic patient glucose as an energy source to prevent precipitation of Wernicke syndrome by depletion of thiamine reserves.

## Treatment Settings for Alcohol Detoxification

Patients with AW can be treated safely and effectively either within a hospital or clinic (i.e., inpatient treatment) or on an ambulatory basis (i.e., outpatient treatment). Although studies have compared the effectiveness of outpatient versus inpatient detoxification, no specific criteria have been rigorously tested.

### Outpatient Treatment

Before the 1980’s, AW was generally treated in an inpatient setting. Today, most detoxifications take place on an outpatient basis. In a review of published studies, Abbott and colleagues (1995) concluded that fewer than 20 percent of patients undergoing AW require admission to an inpatient unit. In addition, more than 70 percent of participants undergoing outpatient detoxification complete the program. In most studies, 50 percent of the patients continued in alcoholism treatment after outpatient detoxification. Most importantly, this review found no reports of serious medical complications among AW outpatients except that one patient suffered a seizure after the start of detoxification.

No specific criteria exist for deciding which patients could benefit from outpatient detoxification. Practical considerations suggest that candidates for outpatient treatment should exhibit only mild to moderate AW symptoms, no medical conditions or severe psychiatric disorders that could complicate the withdrawal process, and no past history of AW seizures or DT’s. In addition, candidates for outpatient detoxification should have a sober significant other to serve as a reliable support person. Ambulatory AW patients should report to their treatment center daily so that the clinician can reassess the patient’s symptoms, the occurrence of medical complications, and ongoing treatment effectiveness.

### Inpatient Treatment

Inpatient detoxification provides the safest setting for the treatment of AW, because it ensures that patients will be carefully monitored and appropriately supported. Compared with outpatient facilities, inpatient clinics may provide better continuity of care for patients who begin alcoholism treatment while in the hospital. In addition, inpatient detoxification separates the patient from alcohol-related social and environmental stimuli that might increase the risk of relapse.

Despite the lack of research-based criteria, certain factors suggest that a patient should receive inpatient treatment (see textbox). These factors include a history of significant AW symptoms, high levels of recent drinking, a history of withdrawal seizures or DT’s, and the co-occurrence of a serious medical or psychiatric illness (Ballenger and Post 1978; Brown et al. 1988).

**Relative Indications for Inpatient Alcohol Detoxification**

- History of severe withdrawal symptoms
- History of alcohol withdrawal seizures or delirium tremens
- Multiple past detoxifications
- Concomitant medical or psychiatric illness
- Recent high levels of alcohol consumption
- Lack of reliable support network
- Pregnancy

### Cost Comparison

The choice of treatment setting for alcohol detoxification has important cost implications. Hayashida and colleagues (1989) found outpatient alcohol detoxification to be considerably less costly than inpatient treatment ($175 to $388 versus $3,319 to $3,665, respectively). To some extent, the higher cost of inpatient treatment reflects the occurrence of more severe symptoms of AW as well as more co-occurring medical problems among hospitalized patients compared with ambulatory patients. However, the safety, efficacy, and cost-effectiveness of outpatient detoxification suggest an important role for this setting in the treatment of mild to moderate AW.

## Nonpharmacological Management

While most clinicians agree that severe AW requires pharmacological treatment, studies suggest that some patients with mild withdrawal symptoms may benefit from supportive care alone. In the context of nonpharmacological therapy, supportive care consists of providing patients with a quiet environment, reduced lighting, limited interpersonal interaction, nutrition and fluids, reassurance, and positive encouragement.

Supportive care does not prevent hallucinations or seizures. In fact, although more than two-thirds of a group of outpatients experiencing mild AW successfully completed detoxification using social support alone, 8 percent had to be referred to an emergency room and 2.5 percent required inpatient admission (Whitfield et al. 1978). However, Shaw and colleagues (1981) found supportive care sufficient treatment for 75 percent of inpatients with no psychiatric or medical problems.

Although these studies suggest that a nonpharmacological approach to treating AW may work for most patients, the data do not provide specific guidance on the selection of treatment types. In addition, supportive care may be more costly, because a greater amount of nursing care may be required during nonpharmacological AW treatment. Until controlled studies of adequate duration and numbers of patients are studied, the role of pharmacological treatment of patients with AW symptoms will continue to be debated.

The most disturbing and perhaps controversial issue regarding nonpharmacological treatment of AW is the concern that failure to medicate may lead to alcohol-induced toxicity to nerve cells (i.e., neurotoxicity), which may increase the patient’s susceptibility to seizures following repeated withdrawals (i.e., kindling) (for further discussion of kindling, see the article by Becker, pp. 25–33) (Ballenger and Post 1978). Therefore, although some withdrawal episodes may appear to be mild enough to be treated without medications, this approach may have long-term deleterious consequences for patients who experience future withdrawal episodes.

## Pharmacological Management

Pharmacological treatment is most frequently employed in moderate to severe AW. Although more than 150 medications have been investigated for the treatment of AW, clinicians disagree on the optimum medications and prescribing schedules. The following review describes some medications that have been recognized as potential treatments for AW.

### Benzodiazepines

Benzodiazepines (BZ’s) are a class of sedative medications widely prescribed to treat anxiety, insomnia, and seizures. Especially in North America, BZ’s are considered by research studies and consensus reports to be the medications of choice to treat AW (American Psychiatric Association Task Force 1989; Institute of Medicine 1990; Anton and Becker 1995; Moskowitz et al. 1983).

Early controlled trials with BZ’s emphasized multiple daily dosing according to a fixed schedule (Kaim et al. 1969). For inpatients in severe AW, a loading procedure has been recommended (Sellers et al. 1983). In this treatment strategy, 10 mg or more of diazepam (Valium^®^) or another long-lasting BZ is administered every hour until either the symptoms are suppressed or the patient becomes excessively sedated. Often only 1 to 2 days of medication are required under this regimen.

Other studies have assessed the need for BZ administration based on the severity of the patient’s symptoms. These assessments have employed a standard AW scale called the Clinical Institute of Withdrawal Assessment for Alcohol, revised (CIWA-Ar) (Saitz et al. 1994). Such studies have found that when the overall dose of BZ’s is reduced, patients suffer less unwanted sedation and are therefore able to participate more readily in other treatment activities. Clearly, the CIWA-Ar is a useful instrument for quantifying AW as well as for guiding the need for medication.

No single BZ appears to be superior to other BZ’s for treating AW (Moskowitz et al. 1983). The selection of a specific BZ for a specific patient has primarily been made on the basis of clinical factors such as the patient’s age; occurrence of prior seizures; and the functional state of the liver, the primary site for the metabolism of BZ’s. In patients with impaired liver function, longer lasting BZ’s may cause problems, ranging from oversedation to incoordination (i.e., ataxia) and confusion. Many alcoholics have liver damage and therefore require medications that are rapidly metabolized.

Recent clinical reviews have stressed the value of short-acting BZ’s, such as oxazepam (Serax^®^) and lorazepam (Ativan^®^) (Gallant 1989). Lorazepam is readily metabolized and is shorter acting than diazepam. O’Brien and colleagues (1983) compared lorazepam and diazepam in patients with moderate AW and found both medications to be equally effective in alleviating AW symptoms, although excessively low blood pressure occurred more commonly in the diazepam-treated patients.

Recently, new practice guidelines were developed by the American Society of Addiction Medicine Working Group on Pharmacological Management of Alcohol Withdrawal (Mayo-Smith 1997). The Working Group reviewed data presented in 134 articles on the treatment of AW published between 1966 and 1995. Based on the review of data, the investigators concluded that BZ’s are “suitable agents for alcohol withdrawal.” All BZ’s appeared equally effective in treating AW symptoms. The Working Group also found that the dose of medication should be individually tailored to suit the symptom severity of each patient. The authors recommended that patients with moderate to severe AW symptoms be treated pharmacologically. Pharmacological treatment should also be administered to patients with a history of withdrawal seizures or in those with comorbid medical illnesses.

### Adrenergic Medications

Adrenergic receptors are specialized proteins on the surface of certain nerve cells. These receptors play an important role in the regulation of the autonomic nervous system and may therefore be expected to influence the occurrence and severity of some withdrawal symptoms. Studies show that medications that alter the function of adrenergic receptors significantly improve symptoms of AW, especially by reducing elevated pulse and blood pressure (Saitz and O’Malley 1997). No evidence indicates, however, that these medications block delirium or seizures. Most reviewers have concluded that adrenergic medications are of value largely as adjuncts to BZ’s in the management of AW. These medications also may be useful in outpatient settings, where the abuse liability of BZ’s by patients is difficult to monitor or prevent and where AW symptoms are generally less severe than among inpatient populations (Anton and Becker 1995).

### Antiseizure Medications

Although in most treatment settings BZ’s are the drugs of choice for uncomplicated AW, nonsedating antiseizure medications may represent desirable alternatives. There are several potential advantages to using antiseizure medications. First, seizures are one of the most serious complications of AW, and the use of an antiseizure medication should decrease the probability of a patient experiencing a seizure. Second, antiseizure medications have been shown to block kindling in brain cells. Third, antiseizure medications do not appear to have abuse potential. Fourth, these medications have been used to treat mood and anxiety disorders, which share some symptoms with AW, including depression, irritability, and anxiety. Fifth, antiseizure medications are generally not as sedating as BZ’s and therefore allow the patient to engage more quickly in alcoholism treatment programs.

In Europe, the antiseizure medications carbamazepine (Tegretol^®^) and valproic acid (Depakene^®^ and others) have been used successfully to treat AW for many years. However, these medications have rarely been used in clinical settings in North America for the treatment of AW. This fact is attributable in part to the reluctance of clinicians to abandon the safe and familiar BZ’s and because most relevant research on the aforementioned medications has been conducted and published outside the United States (Malcolm et al. 1989).

## Treatment of Complicated Withdrawal

### Delirium Tremens

Because DT’s are more likely to occur in patients who have co-occurring medical illnesses, the recognition and aggressive treatment of such illnesses is paramount. The required treatment includes maintaining water and electrolyte balance, correcting metabolic disturbances, and administering medication as appropriate.

The optimum pharmacological therapy for the treatment of DT’s is somewhat controversial. Some clinicians have used BZ’s to decrease autonomic hyperactivity, the risk of AW seizures, and agitation. Despite these beneficial effects, BZ’s may contribute to the aggressive and impulsive behavior and confusion that are elements of DT’s. In addition, withdrawal delirium may develop and persist despite administration of high doses of BZ and adequate control of minor AW symptoms (Hersh et al. 1997).

Antipsychotic medications, such as haloperidol (Haldol^®^), have been used in low doses to treat DT’s. These agents lack the excessive sedation and low blood pressure effects of BZ’s while providing behavioral control. However, antipsychotic medications can cause adverse effects, such as increased susceptibility to seizures, increased restlessness and agitation, and abnormal muscular movements. Clearly, more specific guidelines are needed in the pharmacological treatment of DT’s (Saitz and O’Malley 1997).

### Alcohol Withdrawal Seizures

AW seizures not related to DT’s (i.e., primary AW seizures) usually subside with only supportive treatment. However, because up to one-third of patients with untreated primary seizures subsequently develop DT’s, all primary seizures should be treated. Evidence suggests that for patients who do not have a history of AW seizures, administration of BZ’s should be sufficient to prevent such seizures (Rothstein 1973).

Controversy surrounds the use of the antiepileptic medication phenytoin (Dilantin^®^) in the treatment of AW seizures. Phenytoin does not appear to prevent the occurrence of primary AW seizures. However, phenytoin may be useful in combination with a BZ for preventing an initial seizure in patients who have a history of one or more seizures in their adult life, irrespective of whether any of them were AW seizures (Anton and Becker 1995). More studies are needed in this area, particularly focusing on the efficacy of BZ’s and antiseizure medications, such as carbamazepine and valproic acid, in the treatment and prevention of AW seizures.

## Special Population Issues

Research has not been conducted to determine specific AW strategies for adolescent, geriatric, pregnant, or medically ill populations. No evidence indicates that adolescents with AW require specialized treatment. For the elder population, cumulative years of drinking may lead to more severe withdrawal symptoms (Anton and Becker 1995); the shorter acting BZ’s may be preferable in treating this population, given the increased susceptibility to oversedation in the elderly. Although pregnancy does not appear to increase the occurrence of major withdrawal symptoms, such symptoms may be fatal to both the mother and the fetus. In addition, medications used to treat AW may have adverse effects on the fetus (e.g., congenital malformation). To prevent such risks, treatment with BZ’s should be limited to the minimum amount needed to prevent major complications of AW. (For a discussion of the fetal effects of withdrawal during pregnancy, see the article by Thomas and Riley, pp. 47–53.)

The treatment of AW in the medically ill can present a considerable challenge. These patients are at a higher risk of developing major withdrawal symptoms and may progress to more severe forms of AW, such as DT’s. Concurrent medical conditions should not only be aggressively treated, but should also be anticipated in patients undergoing AW who have been admitted to the hospital for medical or surgical treatment.

## Conclusions

The treatment of patients exhibiting AW has been varied and at times controversial. Although clinicians generally agree that severe AW requires pharmacological intervention, a wide variety of medications have been used. Further uncertainty exists among the treatment community when considering pharmacological treatment of mild to moderate AW, including the preferred treatment setting (i.e., inpatient versus outpatient). While extensive research has been aimed at tackling such issues, a consensus has not yet been reached.

Directions for future research include the continued search for non-BZ treatments for AW. Desirable characteristics of such alternatives would include causing less sedation, exhibiting less interaction with alcohol if both are used concomitantly, and producing antianxiety activity without abuse liability. Additional information is needed concerning the use of clinical scales to quantitate drug effects in AW and clearer specifications on the utility of supportive care in the treatment of AW. Furthermore, considerable research is necessary to further elucidate the role of pharmacotherapy in the treatment of patients who have experienced multiple withdrawal episodes.

## Footnotes

1 A dose of 100 mg thiamine is equivalent to that available in the highest potency nonprescription vitamin B complex supplements. By contrast, the daily requirement is approximately 1.5 mg.
